# Impact of *Plasmodium relictum* Infection on the Colonization Resistance of Bird Gut Microbiota: A Preliminary Study

**DOI:** 10.3390/pathogens13010091

**Published:** 2024-01-20

**Authors:** Justė Aželytė, Apolline Maitre, Lianet Abuin-Denis, Elianne Piloto-Sardiñas, Alejandra Wu-Chuang, Rita Žiegytė, Lourdes Mateos-Hernández, Dasiel Obregón, Alejandro Cabezas-Cruz, Vaidas Palinauskas

**Affiliations:** 1 Nature Research Centre, Akademijos 2, LT-08412 Vilnius, Lithuania; juste.azelyte@gmail.com (J.A.); rita.ziegyte@gamtc.lt (R.Ž.); 2 Anses, National Research Institute for Agriculture, Food and the Environment (INRAE), Ecole Nationale Vétérinaire d’Alfort, UMR BIPAR, Laboratoire de Santé Animale, F-94700 Maisons-Alfort, France; apolline.maitre@anses.fr (A.M.); labuind@gmail.com (L.A.-D.); elianne9409@gmail.com (E.P.-S.); alewch29@gmail.com (A.W.-C.); lourdes.mateos@vet-alfort.fr (L.M.-H.); 3 INRAE, UR 0045 Laboratoire de Recherches Sur Le Développement de L’Elevage (SELMET-LRDE), F-20250 Corte, France; 4 EA 7310, Laboratoire de Virologie, Université de Corse, F-20250 Corte, France; 5 Animal Biotechnology Department, Center for Genetic Engineering and Biotechnology, Avenue 31 between 158 and 190, Havana CU-10600, Cuba; 6 Direction of Animal Health, National Center for Animal and Plant Health, Carretera de Tapaste y Autopista Nacional, Apartado Postal 10, San José de las Lajas CU-32700, Cuba; 7 School of Environmental Sciences, University of Guelph, Guelph, ON N1G 2W1, Canada; dasielogv@gmail.com

**Keywords:** microbiota, avian malaria, colonization resistance, *Plasmodium relictum*

## Abstract

Avian malaria infection has been known to affect host microbiota, but the impact of *Plasmodium* infection on the colonization resistance in bird gut microbiota remains unexplored. This study investigated the dynamics of *Plasmodium relictum* infection in canaries, aiming to explore the hypothesis that microbiota modulation by *P. relictum* would reduce colonization resistance. Canaries were infected with *P. relictum*, while a control group was maintained. The results revealed the presence of *P. relictum* in the blood of all infected canaries. Analysis of the host microbiota showed no significant differences in alpha diversity metrics between infected and control groups. However, significant differences in beta diversity indicated alterations in the microbial taxa composition of infected birds. Differential abundance analysis identified specific taxa with varying prevalence between infected and control groups at different time points. Network analysis demonstrated a decrease in correlations and revealed that *P. relictum* infection compromised the bird microbiota’s ability to resist the removal of taxa but did not affect network robustness with the addition of new nodes. These findings suggest that *P. relictum* infection reduces gut microbiota stability and has an impact on colonization resistance. Understanding these interactions is crucial for developing strategies to enhance colonization resistance and maintain host health in the face of parasitic infections.

## 1. Introduction

Avian malaria, caused by various species of the *Plasmodium* parasite, has been recognized as a significant threat to avian populations worldwide [1]. Among these parasites, *Plasmodium relictum* (genetic lineage SGS1) stands out due to its ability to infect a diverse range of bird species, with more than 300 known hosts documented [2]. As an important pathogen affecting avian health, studying the interactions between *P. relictum* and its avian hosts is crucial for understanding the ecological and evolutionary implications of this parasitic infection.

Colonization resistance, a concept originating from ecological theory, refers to the host’s ability to resist the establishment and proliferation of potential pathogens within its microbiota [3,4,5,6]. The microbiota, consisting of a complex community of microorganisms residing within the host, plays a critical role in maintaining host health and immune homeostasis [7,8]. Over the last few years, research on avian microbiota has markedly increased [8,9,10]. Due to birds inhabiting different environments and adapting to diverse living conditions, their microbiota is complex [11,12]. It can vary between bird species and individuals [11]. Perturbations to the microbiota can have profound effects on the host, potentially influencing disease susceptibility, immune response, and overall well-being [8,13].

While studies have investigated the impact of parasites on the host microbiota in various systems, the specific effects of avian malaria parasites on colonization resistance in birds remain relatively unexplored. Understanding how avian malaria parasites, such as *P. relictum*, influence the colonization resistance of their avian hosts is of great interest to comprehend the ecological dynamics of these infections and their potential implications for avian health.

Several studies have highlighted the importance of the microbiota in mediating host-pathogen interactions and disease outcomes [6,14]. For instance, research conducted on mammalian models has demonstrated that perturbations to the gut microbiota can affect the severity of parasitic infections and influence host immune responses [15,16,17]. Furthermore, the studies conducted by Taniguchi et al. [15] and Mooney et al. [18] revealed a significant correlation between infections of mice with *Plasmodium berghei* and *Plasmodium yoelli*, respectively, and alterations in the abundance of specific bacteria within the gut microbiota. Previous research has primarily focused on analyzing the composition of the microbiota when studying the interactions between avian malaria parasites and the host’s microbiota [19,20,21]. However, it is important to note that the bacterial diversity of the microbiota alone does not fully capture the impact of parasite infection on the host’s microbiota. A recent study by Aželyte et al. [22] investigated the effects of *Plasmodium homocircumflexum* infection on canaries’ microbiota. Interestingly, they found that although the infection did not lead to significant changes in the overall diversity of the microbiota, notable alterations were observed in the bacterial networks within infected canaries at various time points during the infection. Despite advancements in avian microbiota research, it remains unclear whether interactions of *Plasmodium* with resident microbiota affect the response of the gut bacterial community to new invaders or taxa extinction events.

In this study, we aimed to examine the influence of *P. relictum* infection on the colonization resistance of canaries using network analysis. By characterizing the composition and dynamics of the canary gut microbiota in the presence and absence of *P. relictum* infection, we gained insights into the potential interactions between the parasite and the host microbiota. Furthermore, by employing network analysis, specifically node removal and node addition methods, we investigated the resilience and stability of the canary microbiota in the face of *P. relictum* infection. 

Network analysis provides a powerful framework to explore the intricate relationships between individual microbial taxa within a microbiota [23,24], thereby elucidating the mechanisms underlying colonization resistance [25]. By applying node removal and addition methods within the microbiota network, we can assess the influence of specific microbial taxa on network structure [26], connectivity [26,27,28], and resistance [29]. Systematically removing nodes allows us to evaluate the impact on network robustness and identify key taxa that contribute to colonization resistance. Conversely, adding nodes helps us understand how the introduction of certain taxa influences network dynamics and colonization resistance. Through these robustness tests, we can gain insights into the essential microbial players and interactions that drive colonization resistance, providing a comprehensive understanding of the interplay between the host, microbiota, and avian malaria parasite, *P. relictum*.

## 2. Methods

### 2.1. Data Source

**Figure 1:**
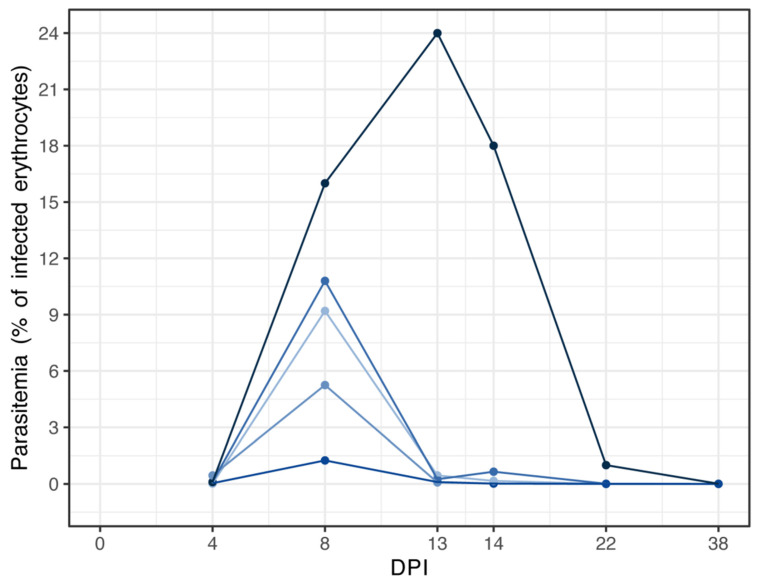
The dynamics of *P. relictum* parasitemia. Individual parasitemia values (% of infected erythrocytes) of *P. relictum* based on microscopy are presented. Different colors represent individual birds. DPI—days post-infection.

To evaluate the impact of avian malaria infection on the bird gut microbiota, we analyzed preliminary data from a previous study on bird infection with *P. relictum* [30]. The study conducted by Aželytė et al. [30] investigated how anti-microbiota vaccination of host birds against commensal bacteria disrupted *P. relictum* sporogonic development by modulating mosquito microbiota. In this study, the 16S rRNA gene sequencing datasets from birds’ fecal material of the *P. relictum* and PBS groups were used. Briefly, a group of 8-month-old canaries was inoculated with meront stages of *P. relictum*, referred to here as the *P. relictum*-infected group. Another group received PBS, referred to here as the control or the uninfected group. The blood was sampled at the indicated DPI (days post-infection) to microscopically calculate the parasitemia (Figure 1). Fecal samples were collected at 22 DPI and 38 DPI. The genomic DNA for microbiome analysis was extracted from feces and sent for amplicon sequencing. Aželytė et al. [30] provide a comprehensive description of the experimental design and procedures.

The 16S rRNA sequences were submitted to the SRA repository under Bioproject No. PRJNA971381. To analyze the raw sequences, we employed the QIIME 2 software package (ver. 2021.4) [31]. The paired-end reads, obtained in fastq files, were processed using the DADA2 pipeline [32]. Taxonomy was assigned to the resulting amplicon sequence variants (ASVs) using a classify-sklearn naïve Bayes taxonomic classifier based on the SILVA database (release 138; [33]). To ensure data quality, we filtered the taxonomic table by removing taxa at the genus level that had a frequency of fewer than 10 reads and were present in less than 3 samples. The resulting data table was then used for microbiota assembly analysis.

### 2.2. Microbiota Diversity, Composition, and Abundance Analyses

To evaluate microbiota diversity and composition of *P. relictum*-infected and control birds at 22 DPI and 38 DPI, alpha and beta diversity of bacterial taxa were analyzed using rarefied ASVs with the q2-diversity plugin in Qiime2 [31]. Microbial richness between the groups was compared with the pairwise Kruskal–Wallis test (*p* < 0.05) using Faith’s phylogenetic diversity [34] and observed features metrics (a measure of taxa inventory), while the evenness was calculated by Pielou’s index [35]. The beta diversity between the groups was assessed using the Bray–Curtis dissimilarity index [36] with a PERMANOVA test (*p* < 0.05). Beta dispersion was calculated using the betadisper function of the Vegan package implemented in the R program (ver. 4.1.3) [37]. The dispersion was compared between the groups using a PERMANOVA test (*p* < 0.05). 

Differences in taxa abundance between the groups were calculated using the ANOVA-like differential expression package ‘ALDEx2’ [38] in the R program (ver. 4.1.3) [37]. This method utilizes a centered log ratio (clr) transformation based on the geometric mean of read counts in the sample to measure relative abundance [39]. The comparisons were performed with a *t*-test (*p* ≤ 0.05). The numbers of shared taxa in the microbiota of *P. relictum*-infected and control groups were visualized using Venn diagrams implemented in the online tool (http://bioinformatics.psb.ugent.be/webtools/Venn/; accessed on 10 May 2023). 

By employing these methods and tools, this study aimed to comprehensively evaluate and compare the microbial diversity, composition, richness, evenness, beta diversity, dispersion, and taxa abundance between the *P. relictum*-infected and control groups of birds at two time points (22 DPI and 38 DPI).

### 2.3. Bacterial Co-Occurrence Networks

Co-occurrence microbial networks were constructed to visually represent the assembly of microbial communities under different conditions. These networks were based on taxonomic profiles at the genera level. In the network, nodes represent bacterial taxa, and the edges indicate significant positive (weight > 0.75) or negative (weight < −0.75) co-occurrence interactions between the nodes. The Sparse Correlations for Compositional data (SparCC) method [40] implemented in the R program (ver. 4.1.3) [37] was used to analyze constructed networks. Gephi 0.9.5 [41] software was employed for network visualization and measuring various topological features of each group, such as the number of nodes and edges, network diameter, average degree, weighted degree, average path length, modularity, and number of modules.

### 2.4. Comparative Network Analysis and Robustness

The microbial networks were compared between the conditions using various functions of the NetCoMi package [42] in the R program (ver. 4.1.3) [37]. To assess the similarities between networks based on shared nodes and edges, an association analysis was conducted. The degree of similarity between networks increases as the number of shared nodes and edges increases. For the comparison of the most central nodes in the networks, two *p*-values, P(J ≤ j) and P(J ≥ j), were calculated for each Jaccard index. These *p*-values represent the probability that the observed Jaccard index (J) value is either “less than or equal to” or “greater than or equal to” the expected Jaccard value at random (j). Differences were considered significant when *p* < 0.05. 

The core association network (CAN) analysis function of the NetCoMi package [42] was used to evaluate the common nodes and edges between two different networks. The core of the control and *P. relictum*-infected groups’ networks was determined at two different time points using the Anuran toolbox [43] with default parameters. This analysis was conducted in the Anaconda Python environment (ver. 3.9.17) [44]. 

To test the robustness of the network to node removal, the Network Strengths and Weaknesses Analysis (NetSwan) package (ver. 0.1) was employed [45]. Various node removal attacks, including random, betweenness centrality, degree, and cascading, were performed to assess network tolerance based on connectivity loss. The standard error for loss of connectivity was calculated, considering variability, using a threshold of 0.975. The igraph package was utilized for network analysis and visualization [46,47].

The robustness of microbial networks to node addition was assessed using the network analysis and visualization package [48]. Nodes were incrementally added in sections ranging from 100 to 1000, and network connectivity was measured based on the degree metric of the largest connected component (LCC) and average path length. A Wilcoxon signed-rank test was conducted to calculate *p*-values for LCC and average path length. The *p*-values were adjusted using the Benjamini–Hochberg (BH) method to control the false discovery rate. Additionally, bootstrapping was performed to obtain confidence intervals for the variables. Significance was determined at a threshold of *p* < 0.05.

## 3. Results

### 3.1. Dynamics of Plasmodium relictum Infection

An experiment was conducted over a duration of 38 days to investigate the effects of *P. relictum* infection. A group of five canaries received an inoculation of infected blood containing *P. relictum* meronts, while another group of three canaries was injected with parasite-free PBS and served as the control. Four days after the infection, *P. relictum* was detected in the peripheral blood of all infected canaries (Figure 1). The highest parasitemia, which is measured by the percentage of infected erythrocytes, was observed at 8 days post-infection (DPI) in four *P. relictum*-infected birds, with an average of 6.6 ± 4.3% (mean ± SD). One bird reached the peak parasitemia of 24% at 13 DPI. Three birds showed low parasitemia in their peripheral blood at 22 DPI and 38 DPI (22 DPI—0.2 ± 0.4%; 38 DPI—0.004 ± 0.004%) (Figure 1).

### 3.2. The Impact of Plasmodium relictum Infection on Host Microbiota Diversity and Composition

**Figure 2:**
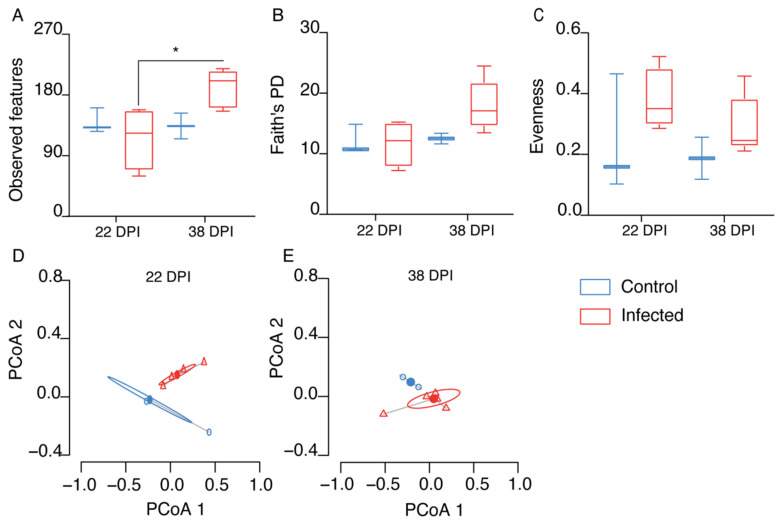
Comparison of microbial diversity to assess the impact of *P. relictum* infection on the microbiota in birds between infected and control groups at 22 and 38 days post-infection (DPI). (**A**) Observed features, (**B**) Faith’s phylogenetic diversity (PD), and (**C**) Pielou’s evenness index. Comparison of beta–diversity with Bray–Curtis dissimilarity index for infected (triangle) and control (circle) groups at 22 (**D**) and 38 DPI (**E**), represented in PCoA plot obtained by Betadisper function. * *p* < 0.05.

We conducted an analysis of bird microbiota from fecal samples using amplicon sequence variants (ASV) to examine the impact of *P. relictum* infection on the host microbiota. We compared infected and control groups at two time points: 22 DPI and 38 DPI. To measure the diversity, we calculated three alpha diversity metrics, namely observed features (Figure 2A), phylogenetic diversity (Faith’s phylogenetic diversity index) (Figure 2B), and evenness (Pielou’s index) (Figure 2C). Surprisingly, there were no significant differences in these metrics between the infected and control groups (Kruskal–Wallis test, *p* > 0.05). However, we found significant differences in the observed features within the infected group between 22 DPI and 38 DPI (Figure 2A).

To assess microbial community composition, we used the Bray–Curtis index. We found a significant difference in the composition of the microbiota between the infected and control groups (PERMANOVA, *p* = 0.007; F = 4.604; Figure 2D,E), while beta dispersion showed no significant differences (PERMANOVA test, *p* > 0.05; Figure 2D,E).

Next, we conducted a differential abundance analysis to identify changes in specific taxa between *P. relictum*-infected and control birds. At 22 DPI, we observed a significantly higher abundance of three taxa in the microbiota of infected birds, while the control group had a higher abundance of seven taxa (Table S1, Figure S1). At 38 DPI, the control group had 23 taxa with a significantly increased abundance compared to the *P. relictum*-infected group. Interestingly, the bacterial taxa with significant differences in abundance were different at 22 DPI and 38 DPI and unique to the groups (Table S1, Figure S1).

### 3.3. Changes in the Microbiota Assembly Due to Plasmodium relictum Infection 

**Figure 3:**
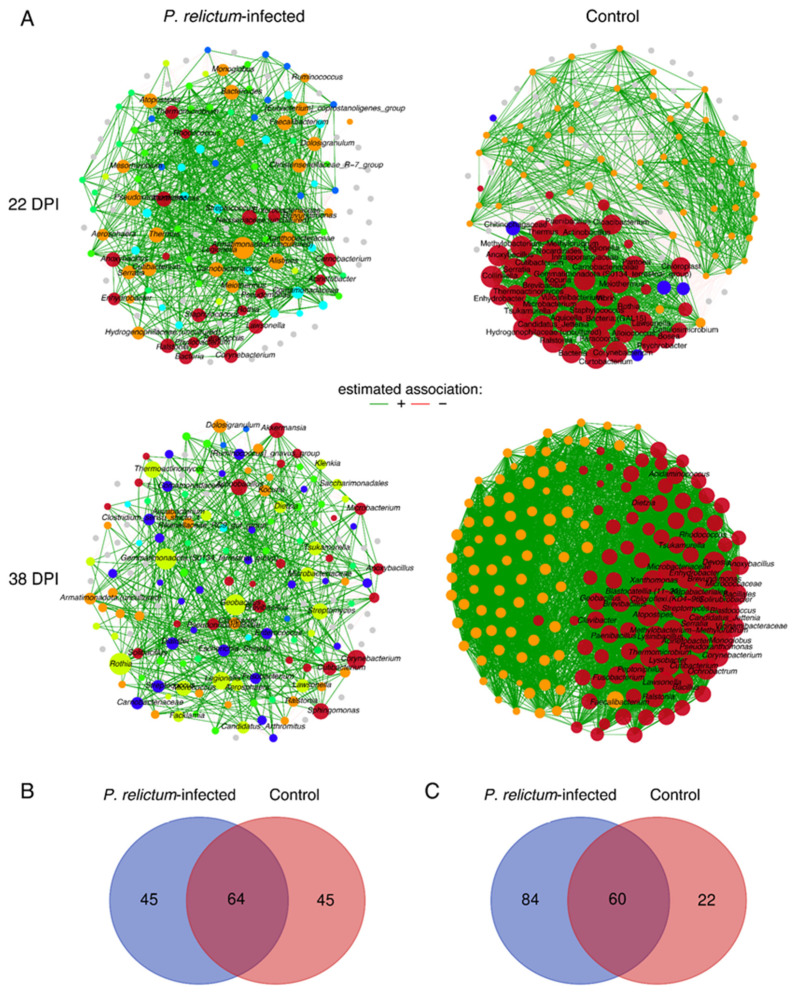
Microbial community assemblies in *P. relictum*-infected and uninfected birds. Co-occurrence networks (**A**) were extrapolated from the microbiota of *P. relictum*-infected and control birds at 22 DPI and 38 DPI. Bacterial taxa with at least one connection are symbolized by nodes, whilst connected edges represent a significant correlation between them. The width of the edges corresponds to the level of co-occurrence correlation (SparCC, weight ≥ 0.5 or ≤−0.5). Green edges represent positive correlations. The colors of nodes specify clusters and modules in which taxa occur. The size of nodes is related to their eigenvector centrality. Venn diagrams displaying the number of shared and unique taxa detected within *P. relictum*-infected and control birds at 22 DPI (**B**) and 38 DPI (**C**).

We compared the microbiota structure in uninfected and *P. relictum*-infected birds using bacterial co-occurrence networks. In the infected group, we observed fewer correlations between nodes compared to the control group at both 22 DPI and 38 DPI (Figure 3A). The correlation patterns between nodes in the two groups changed differently from 22 DPI to 38 DPI. At 22 DPI, both networks had the same number of nodes (Table 1). However, Venn analysis revealed that out of a total of 154 nodes, 64 (41.6%) were shared between the control and infected groups, with each group having 45 (29.2%) unique nodes (Figure 3B). From 22 DPI to 38 DPI, the number of nodes in the infected group’s network increased, while the number of nodes declined in the control group. The bacterial network of infected birds comprised 84 (50.6%) unique nodes and 60 (36.1%) shared nodes with the control group, which, in turn, had 22 (33.3%) unique genera (Figure 3C). However, the number of correlations in the control group’s network was notably higher at both time points compared to the infected birds (Table 1).

When comparing the modularity in the networks—referring to a degree of division into distinct communities—we found that this parameter was higher in the control group at 22 DPI compared to the infected group, while at 38 DPI, the pattern was inverted. Notably, the microbiota of the infected group showed greater interconnectedness with the presence of several distinct clusters (Figure 3A). In the control group’s networks, both at 22 DPI and 38 DPI, a major cluster with nodes with the highest values of eigenvector centrality was observed (Figure 3A, red nodes), but this pattern was not observed in the infected group’s networks. Notably, only positive correlations were shared between the groups, while all negative correlations were specific to each group.

The observed Jaccard values for comparing degree and betweenness centrality between networks of *P. relictum*-infected and control birds were significantly lower than expected by random at 22 DPI. However, there were no significant differences in hub taxa, closeness, and eigenvector centrality (Table S2). At 38 DPI, all centrality measures showed significantly lower observed Jaccard values between the groups compared to random expectations. These significant values were all below 0.3, indicating a low similarity in centrality measures’ distribution in the microbiota of *P. relictum*-infected and control birds (Table S2).

**Figure 4:**
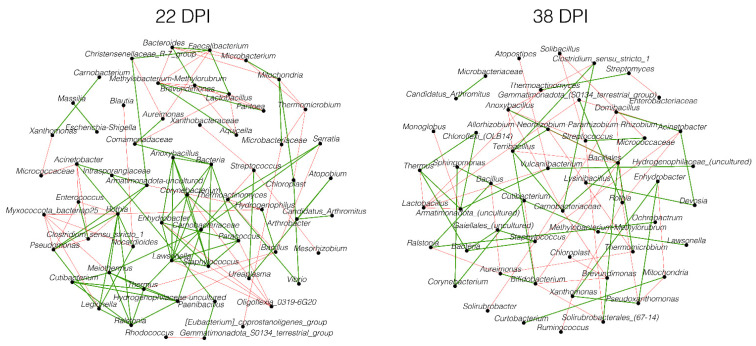
Core association networks inferred between *P. relictum*-infected and control groups at 22 DPI and 38 DPI. Positive (green) or negative (red) correlations are shown by the color of the edges. Nodes represent bacterial taxa.

Despite the differences in the community assembly, the analysis of the core association network (CAN) revealed 62 core-associated nodes between the infected and control groups at 22 DPI (Figure 4). Among these core-associated nodes, we determined 72 (59% of the total 122) positive edges and 50 (41% of the total 122) negative edges, which were common in both groups. At 38 DPI, the core-associated network consisted of 52 nodes, with 38 (50% of the total 76) positive and 38 (50% of the total 76) negative edges.

### 3.4. Network Robustness

**Figure 5:**
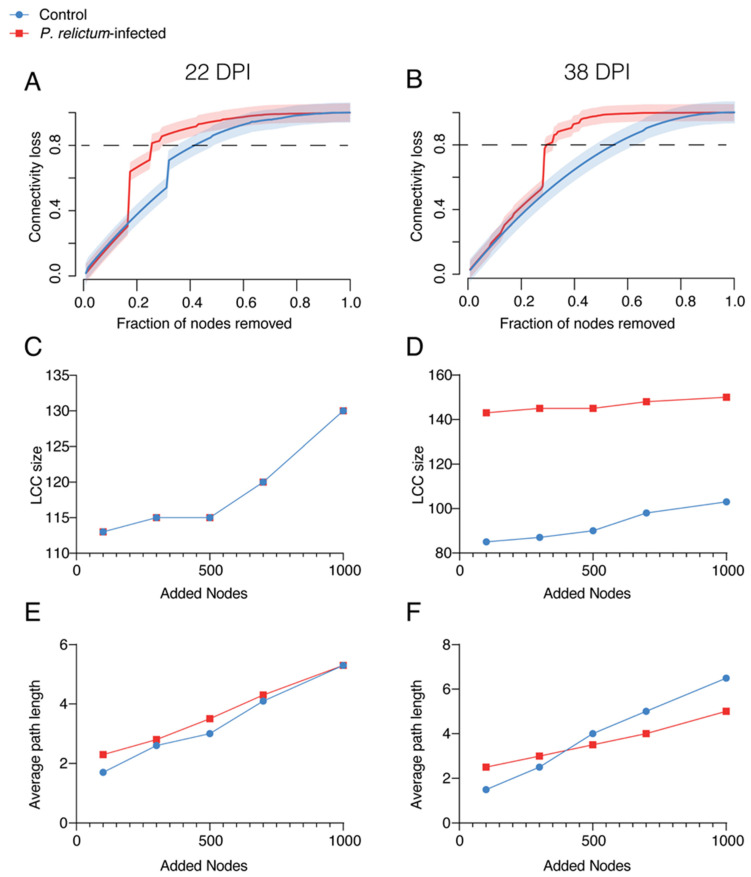
(**A**,**B**) Network robustness to node removal with cascading attack. Values of connectivity loss in *P. relictum*-infected (red) and control (blue) birds at 22 DPI and 38 DPI were compared. (**C**–**F**) Comparison of network robustness to node addition. The values of the largest connected component (LCC) (**C**,**D**) and average path length (**E**,**F**) are presented.

To assess the robustness of the bird microbiota networks during infection and in a healthy state at different time points, we tested the loss of connectivity due to node removal using different attack methods: direct, cascading, degree, or random. The results showed that the cascading method had the highest impact on network connectivity in infected birds compared to the control group. The most notable difference in network tolerance to perturbations between infected and control birds was observed at 80% connectivity loss. At 22 DPI, the infected group required the removal of 0.25 fraction of nodes in the network, while the control group required 0.42 to achieve the specified loss of connectivity (Figure 5A). At 38 DPI, the fraction of nodes removed increased to 0.30 for infected birds and 0.55 for the control group to achieve the same effect on the network (Figure 5B). Other node removal methods did not show a visible difference in connectivity loss between the groups (Figure S2).

To compare the network stability of infected and control groups, we examined how the addition of new nodes affected network connectivity. The results revealed that the largest connected component (LCC) increased in both groups after adding more than 500 nodes to the networks at 22 DPI (Figure 5C,D). At 38 DPI, the infected group initially exhibited a high value of LCC, which did not significantly change even after adding more nodes, indicating a high stability of the network (Figure 5C). In contrast, the LCC in the control group remained relatively low compared to the infected group, with a minor increase when more nodes were added, suggesting less tolerance to changes in the microbiota (Figure 5D).

A similar pattern of changes in the average path length was observed in response to node additions in both infected and control groups (Figure 5E,F). Adding nodes to the networks increased the average path length in both groups at 22 DPI and 38 DPI (Figure 5E,F). However, at 38 DPI, the effects on the infected group’s network were less pronounced compared to the control group.

## 4. Discussion

The influence of malaria parasites on the modulation of the microbiota has been established in previous studies [21,22]. In this study, we put forth the hypothesis that *P. relictum* plays a pivotal role in reshaping the microbial community within the host through intricate microbe–host interactions. Consequently, this process may result in a decreased ability of the microbiota to resist colonization. To investigate this phenomenon, we adopted a network-based approach to evaluate the effects of *P. relictum* on the composition of the microbiota in canaries.

We found no significant differences in alpha diversity metrics between the avian malaria parasite *P. relictum*-infected and control groups. This discovery aligns with the studies of Aželyte et al. [22] and Rohrer et al. [21], where an infection caused by avian malaria parasites did not greatly affect the alpha and beta diversity of the avian host. These results suggest that avian malaria infections have a negligible impact on the diversity of the host microbiota. In contrast, it is reported that the non-human primate and murine malaria parasites can significantly reduce alpha diversity [15,18,49]. Several studies showed that lower diversity of microbial species within the microbiota has been linked to reduced colonization resistance in other systems [50,51,52]. The current findings on the impact of avian *Plasmodium* on host-microbiota variety imply that it has a minimal impact on colonization resistance.

However, this study revealed noteworthy disparities in beta diversity between the *P. relictum*-infected and control groups, suggesting alterations in the microbiota composition of infected birds. This contrasts with the findings of Aželytė et al. [22] and Rohrer et al. [21], who reported no divergence in beta diversity between *Plasmodium*-infected birds and the control group. The observed changes in microbial composition in this study imply that *P. relictum* infection disturbs the normal equilibrium of bacterial taxa within the canaries’ microbiota. Furthermore, upon analyzing differential abundance, we identified specific taxa with higher abundance, mostly in uninfected birds. These findings align with the studies conducted by Rohrer et al. [21], which observed fewer taxa with increased abundance in *Plasmodium*-infected birds compared to the control. Importantly, our findings are similar to the results of Aželytė et al. [22], who found no significant variations in taxa abundance between infected and uninfected birds at different time points. It is noteworthy that our results from the experiment conducted under controlled conditions are comparable to the study by Rohrer et al. [21], which investigated wild birds. Although the studies showed that microbiota composition is influenced by genetic background and other environmental factors [13,53], the alterations caused by infection can be recognized. The lack of change in alpha diversity suggests that overall microbial richness and evenness may remain relatively stable despite the presence of the parasite. However, the observed shifts in composition and abundance of commensal bacteria highlight the potential for specific taxa to be affected by the infection, which may have important implications for colonization resistance. Spragge et al. [52] showed that colonization resistance is not dependent on a single species but rather on the associations of multiple bacteria cohesively living in a community. These findings suggest that while the overall diversity of the microbiota may not be impacted, specific microbial communities crucial for colonization resistance could be compromised by *P. relictum* infection.

In agreement with the findings of others in different parasites [14,22], this study reveals a decrease in network correlations, indicating that infection with *P. relictum* is linked to a reduction in the complexity and connectivity of the microbiota structure. As in *P. homocircumflexum* [22], we observed distinct changes in the correlation patterns between infected and control groups over time, suggesting that the infection dynamically affects the interactions among bacterial species within the community assembly, leading to alterations in the network structure. Furthermore, the varying proportions of unique and shared nodes between the groups indicate that *P. relictum*-related changes in the composition of the bacterial community result in changes in network membership. However, intriguingly, our CAN results imply that certain bacterial taxa maintain consistent associations despite the infection, while other associations may be specific to each group.

This disruption in community assembly may weaken the overall stability and resilience of the microbiota, potentially compromising its ability to resist colonization by pathogens. Disruption can occur due to drug administration, such as anthelmintics [54] or antibiotics [55], or pathogen infection [56], and these have been shown to affect colonization resistance [25]. For example, the administration of anthelmintic drugs significantly altered the microbial community of Welsh ponies, causing instability and disrupting the time-dependent network of interactions [54]. This disruption had long-term effects on microbial resilience [54]. Antibiotic use also disrupts the balance between host and microbiota, leading to *Clostridium difficile* infection [55]. Moreover, acute infection by *Yersinia* can induce long-term immune and microbiota changes, ultimately resulting in chronic inflammatory disease [56]. 

Furthermore, changes in centrality measures may impact the key microbial species involved in colonization resistance, potentially compromising the microbiota’s ability to resist pathogen colonization. An example of this can be seen in the protective role of *Enterococcus faecalis* against *Staphylococcus aureus* in *Caenorhabditis elegans* [57], where *E. faecalis* becomes a keystone taxon in the nematode’s microbiota [58]. Our findings on network robustness support the notion that *Plasmodium* infection has an impact on colonization resistance. Infected birds experienced greater disruption of network connectivity when taxa were removed, but their networks also showed higher stability and less pronounced changes after node additions.

## 5. Conclusions

In conclusion, this study on avian malaria parasite *P. relictum* infection suggests that, while overall microbial diversity remains stable, specific bacterial communities crucial for colonization resistance may be compromised. The infection disrupts the composition and connectivity of the microbiota, potentially weakening its ability to resist pathogen colonization. These findings have broader implications beyond avian malaria infections. Disruptions in community assembly, alterations in network structure, and changes in microbial abundances have been linked to compromised colonization resistance in other systems. Similar disruptions have been observed due to drug administration or pathogen infections, highlighting the importance of maintaining a healthy and stable microbiota for the host defense. Further research is needed to understand the intricate mechanisms underlying these interactions and develop strategies to enhance colonization resistance and host health.

## Figures and Tables

**Table 1:** Topological parameters of co-occurrence networks.

Network Features	Uninfected		*P. relictum*-Infected	
	22 DPI	38 DPI	22 DPI	38 DPI
Nodes	109	82	109	139
Edges	2943	3235	1201	904
Positive	1482 (50.36%)	1558 (48.16%)	624 (51.96%)	461 (51%)
Negative	1461 (49.64%)	1677 (51.84%)	577 (48.04%)	443 (49%)
Network diameter	3	2	4	5
Average degree	54	78.902	22.037	13.007
Weighted degree	0.638	−2.286	0.877	0.257
Average path length	1.509	1.026	2.238	2.673
Modularity	19.062	−20.16	9.353	17.473
Number of modules	3	2	3	7
Average clustering coefficient	0.759	0.978	0.667	0.539

## Data Availability

The datasets generated and analyzed in this study are available on the SRA repository under Bioproject No. PRJNA971381.

## Supplementary Materials

The following supporting information can be downloaded at https://www.mdpi.com/article/10.3390/pathogens13010091/s1. Figure S1: Heatmaps representing the relative abundance (expressed as centered log ratio) of taxa with significant differences between uninfected and *P. relictum*-infected birds at 22 DPI (A) and 38 DPI (B). Figure S2: Network tolerance to node removal. The resistance of the networks to directed, degree, cascading, and random attacks in control and *P. relictum*-infected groups at 22 DPI and 38 DPI. Table S1: List of unique and differentially abundant taxa identified by the ALDEx2 method between the gut microbiota of infected and uninfected birds at 22 DPI and 38 DPI. Table S2: Jaccard indexes of local centrality measures. The two *p*-values, P(J ≤ j) and P(J ≥ j), for each Jaccard’s index were added.

## References

[1] Valkiūnas G. (2005). Avian Malaria Parasites and Other Haemosporidia.

[2] Ducarmon Q.R., Zwittink R.D., Hornung B.V.H., van Schaik W., Young V.B., Kuijper E.J. (2019). Gut Microbiota and Colonization Resistance against Bacterial Enteric Infection. Microbiol. Mol. Biol. Rev..

[3] Karita Y., Limmer D.T., Hallatschek O. (2022). Scale-Dependent Tipping Points of Bacterial Colonization Resistance. Proc. Natl. Acad. Sci. USA.

[4] Mullineaux-Sanders C., Suez J., Elinav E., Frankel G. (2018). Sieving through Gut Models of Colonization Resistance. Nat. Microbiol..

[5] Stacy A., Andrade-Oliveira V., McCulloch J.A., Hild B., Oh J.H., Perez-Chaparro P.J., Sim C.K., Lim A.I., Link V.M., Enamorado M. (2021). Infection Trains the Host for Microbiota-Enhanced Resistance to Pathogens. Cell.

[6] Martínez-de la Puente J., Santiago-Alarcon D., Palinauskas V., Bensch S. (2021). Plasmodium Relictum. Trends Parasitol..

[7] Chen Y., Li H. (2022). Avian Leukosis Virus Subgroup J Infection Influences the Gut Microbiota Composition in Huiyang Bearded Chickens. Lett. Appl. Microbiol..

[8] Sun F., Chen J., Liu K., Tang M., Yang Y. (2022). The Avian Gut Microbiota: Diversity, Influencing Factors, and Future Directions. Front. Microbiol..

[9] Waite D.W., Taylor M.W. (2015). Exploring the Avian Gut Microbiota: Current Trends and Future Directions. Front. Microbiol..

[10] Palinauskas V., Mateos-Hernandez L., Wu-Chuang A., de la Fuente J., Aželytė J., Obregon D., Cabezas-Cruz A. (2022). Exploring the Ecological Implications of Microbiota Diversity in Birds: Natural Barriers against Avian Malaria. Front. Immunol..

[11] Cisek A.A., Binek M. (2014). Chicken Intestinal Microbiota Function with a Special Emphasis on the Role of Probiotic Bacteria. Pol. J. Vet. Sci..

[12] Hird S.M., Sánchez C., Carstens B.C., Brumfield R.T. (2015). Comparative Gut Microbiota of 59 Neotropical Bird Species. Front. Microbiol..

[13] Grond K., Santo Domingo J.W., Lanctot R.B., Jumpponen A., Bentzen R.L., Boldenow M.L., Brown S.C., Casler B., Cunningham J.A., Doll A.C. (2019). Composition and Drivers of Gut Microbial Communities in Arctic-Breeding Shorebirds. Front. Microbiol..

[14] Mammeri M., Obregón D.A., Chevillot A., Polack B., Julien C., Pollet T., Cabezas-Cruz A., Adjou K.T. (2020). Cryptosporidium Parvum Infection Depletes Butyrate Producer Bacteria in Goat Kid Microbiome. Front. Microbiol..

[15] Taniguchi T., Miyauchi E., Nakamura S., Hirai M., Suzue K., Imai T., Nomura T., Handa T., Okada H., Shimokawa C. (2015). Plasmodium Berghei ANKA Causes Intestinal Malaria Associated with Dysbiosis. Sci. Rep..

[16] Stough J.M.A., Dearth S.P., Denny J.E., LeCleir G.R., Schmidt N.W., Campagna S.R., Wilhelm S.W. (2016). Functional Characteristics of the Gut Microbiome in C57BL/6 Mice Differentially Susceptible to *Plasmodium yoelii*. Front. Microbiol..

[17] Yilmaz B., Portugal S., Tran T.M., Gozzelino R., Ramos S., Gomes J., Regalado A., Cowan P.J., d’Apice A.J.F., Chong A.S. (2014). Gut Microbiota Elicits a Protective Immune Response against Malaria Transmission. Cell.

[18] Mooney J.P., Lokken K.L., Byndloss M.X., George M.D., Velazquez E.M., Faber F., Butler B.P., Walker G.T., Ali M.M., Potts R. (2015). Inflammation-Associated Alterations to the Intestinal Microbiota Reduce Colonization Resistance against Non-Typhoidal Salmonella during Concurrent Malaria Parasite Infection. Sci. Rep..

[19] Navine A.K., Paxton K.L., Paxton E.H., Hart P.J., Foster J.T., McInerney N., Fleischer R.C., Videvall E. (2023). Microbiomes Associated with Avian Malaria Survival Differ between Susceptible Hawaiian Honeycreepers and Sympatric Malaria-resistant Introduced Birds. Mol. Ecol..

[20] Videvall E., Marzal A., Magallanes S., Fleischer R.C., Espinoza K., García-Longoria L. (2021). The Uropygial Gland Microbiome of House Sparrows with Malaria Infection. J. Avian Biol..

[21] Rohrer S.D., Robertson B.Q., Chubiz L.M., Parker P.G. (2023). Gut Microbiome Composition Associated with *Plasmodium* Infection in the Eurasian Tree Sparrow. J. Avian Biol..

[22] Aželytė J., Wu-Chuang A., Maitre A., Žiegytė R., Mateos-Hernández L., Obregón D., Palinauskas V., Cabezas-Cruz A. (2023). Avian Malaria Parasites Modulate Gut Microbiome Assembly in Canaries. Microorganisms.

[23] Faust K., Raes J. (2012). Microbial Interactions: From Networks to Models. Nat. Rev. Microbiol..

[24] Röttjers L., Faust K. (2018). From Hairballs to Hypotheses–Biological Insights from Microbial Networks. FEMS Microbiol. Rev..

[25] Shade A., Peter H., Allison S.D., Baho D.L., Berga M., Bürgmann H., Huber D.H., Langenheder S., Lennon J.T., Martiny J.B.H. (2012). Fundamentals of Microbial Community Resistance and Resilience. Front. Microbio.

[26] Maitre A., Wu-Chuang A., Mateos-Hernández L., Piloto-Sardiñas E., Foucault-Simonin A., Cicculli V., Moutailler S., Paoli J., Falchi A., Obregón D. (2023). Rickettsial Pathogens Drive Microbiota Assembly in *Hyalomma Marginatum* and *Rhipicephalus Bursa* Ticks. Mol. Ecol..

[27] Maitre A., Wu-Chuang A., Mateos-Hernández L., Foucault-Simonin A., Moutailler S., Paoli J.-C., Falchi A., Díaz-Sánchez A.A., Banović P., Obregón D. (2022). Rickettsia Helvetica Infection Is Associated with Microbiome Modulation in Ixodes Ricinus Collected from Humans in Serbia. Sci. Rep..

[28] Mateos-Hernández L., Obregón D., Wu-Chuang A., Maye J., Bornères J., Versillé N., de la Fuente J., Díaz-Sánchez S., Bermúdez-Humarán L.G., Torres-Maravilla E. (2021). Anti-Microbiota Vaccines Modulate the Tick Microbiome in a Taxon-Specific Manner. Front. Immunol..

[29] Estrada-Peña A., Cabezas-Cruz A., Obregón D. (2020). Resistance of Tick Gut Microbiome to Anti-Tick Vaccines, Pathogen Infection and Antimicrobial Peptides. Pathogens.

[30] Aželytė J., Wu-Chuang A., Žiegytė R., Platonova E., Mateos-Hernandez L., Maye J., Obregon D., Palinauskas V., Cabezas-Cruz A. (2022). Anti-Microbiota Vaccine Reduces Avian Malaria Infection within Mosquito Vectors. Front. Immunol..

[31] Bolyen E., Rideout J.R., Dillon M.R., Bokulich N.A., Abnet C.C., Al-Ghalith G.A., Alexander H., Alm E.J., Arumugam M., Asnicar F. (2019). Reproducible, Interactive, Scalable and Extensible Microbiome Data Science Using QIIME 2. Nat. Biotechnol..

[32] Callahan B.J., McMurdie P.J., Rosen M.J., Han A.W., Johnson A.J.A., Holmes S.P. (2016). DADA2: High-Resolution Sample Inference from Illumina Amplicon Data. Nat. Methods.

[33] Yarza P., Yilmaz P., Pruesse E., Glöckner F.O., Ludwig W., Schleifer K.-H., Whitman W.B., Euzéby J., Amann R., Rosselló-Móra R. (2014). Uniting the Classification of Cultured and Uncultured Bacteria and Archaea Using 16S rRNA Gene Sequences. Nat. Rev. Microbiol..

[34] Faith D.P. (1992). Conservation Evaluation and Phylogenetic Diversity. Biol. Conserv..

[35] Bray J.R., Curtis J.T. (1957). An Ordination of the Upland Forest Communities of Southern Wisconsin. Ecol. Monogr..

[36] Pielou E.C. (1966). The Measurement of Diversity in Different Types of Biological Collections. J. Theor. Biol..

[37] R Core Team (2021). R: A Language and Environment for Statistical Computing.

[38] Gloor G.B., Macklaim J.M., Fernandes A.D. (2016). Displaying Variation in Large Datasets: Plotting a Visual Summary of Effect Sizes. J. Comput. Graph. Stat..

[39] Aitchison J. (1986). The Statistical Analysis of Compositional Data.

[40] Friedman J., Alm E.J. (2012). Inferring Correlation Networks from Genomic Survey Data. PLoS Comput. Biol..

[41] Bastian M., Heymann S., Jacomy M. (2009). Gephi: An Open Source Software for Exploring and Manipulating Networks. ICWSM.

[42] Peschel S., Müller C.L., von Mutius E., Boulesteix A.-L., Depner M. (2021). NetCoMi: Network Construction and Comparison for Microbiome Data in R. Brief. Bioinform..

[43] Röttjers L., Vandeputte D., Raes J., Faust K. (2021). Null-Model-Based Network Comparison Reveals Core Associations. ISME Commun..

[44] Anaconda Software Distribution (2023). Anaconda Documentation. Anaconda Inc.. https://docs.anaconda.com/.

[45] Lhomme S. (2015). Analyse Spatiale de La Structure Des Réseaux Techniques Dans Un Contexte de Risques. Cybergeo.

[46] Csárdi G., Nepusz T. (2006). The Igraph Software Package for Complex Network Research. InterJournal Complex Syst..

[47] Csárdi G., Nepusz T., Müller K., Horvát S., Traag V., Zanini F., Noom D. Igraph for R: R Interface of the Igraph Library for Graph Theory and Network Analysis 2023. Zenodo. https://CRAN.R-project.org/package=igraph.

[48] Freitas S., Yang D., Kumar S., Tong H., Chau D.H. (2020). Evaluating Graph Vulnerability and Robustness Using TIGER. arXiv.

[49] Farinella D.N., Kaur S., Tran V., Cabrera-Mora M., Joyner C.J., Lapp S.A., Pakala S.B., Nural M.V., DeBarry J.D., Kissinger J.C. (2023). Malaria Disrupts the Rhesus Macaque Gut Microbiome. Front. Cell. Infect. Microbiol..

[50] Robinson C.J., Schloss P., Ramos Y., Raffa K., Handelsman J. (2010). Robustness of the Bacterial Community in the Cabbage White Butterfly Larval Midgut. Microb. Ecol..

[51] Shea K. (2002). Community Ecology Theory as a Framework for Biological Invasions. Trends Ecol. Evol..

[52] Spragge F., Bakkeren E., Jahn M.T., Araujo E.B.N., Pearson C.F., Wang X., Pankhurst L., Cunrath O., Foster K.R. (2023). Microbiome Diversity Protects against Pathogens by Nutrient Blocking. Science.

[53] Schokker D., Veninga G., Vastenhouw S.A., Bossers A., De Bree F.M., Kaal-Lansbergen L.M.T.E., Rebel J.M.J., Smits M.A. (2015). Early Life Microbial Colonization of the Gut and Intestinal Development Differ between Genetically Divergent Broiler Lines. BMC Genom..

[54] Boisseau M., Dhorne-Pollet S., Bars-Cortina D., Courtot É., Serreau D., Annonay G., Lluch J., Gesbert A., Reigner F., Sallé G. (2023). Species Interactions, Stability, and Resilience of the Gut Microbiota—Helminth Assemblage in Horses. iScience.

[55] Britton R.A., Young V.B. (2012). Interaction between the Intestinal Microbiota and Host in Clostridium Difficile Colonization Resistance. Trends Microbiol..

[56] Kamdar K., Khakpour S., Chen J., Leone V., Brulc J., Mangatu T., Antonopoulos D.A., Chang E.B., Kahn S.A., Kirschner B.S. (2016). Genetic and Metabolic Signals during Acute Enteric Bacterial Infection Alter the Microbiota and Drive Progression to Chronic Inflammatory Disease. Cell Host Microbe.

[57] King K.C., Brockhurst M.A., Vasieva O., Paterson S., Betts A., Ford S.A., Frost C.L., Horsburgh M.J., Haldenby S., Hurst G.D. (2016). Rapid Evolution of Microbe-Mediated Protection against Pathogens in a Worm Host. ISME J..

[58] Wu-Chuang A., Bates K.A., Obregon D., Estrada-Peña A., King K.C., Cabezas-Cruz A. (2022). Rapid Evolution of a Novel Protective Symbiont into Keystone Taxon in Caenorhabditis Elegans Microbiota. Sci. Rep..

