# Supplementary material for: Impact of Plasmodium relictum Infection on the Colonization Resistance of Bird Gut Microbiota: A Preliminary Study
Source: Pathogens. 2024 Jan 20;13(1):91. doi: 10.3390/pathogens13010091 (PMC10819382; doi:10.3390/pathogens13010091)
Supplement: Supplementary file 1 [file pathogens-13-00091-s001.zip › Table S1.pdf]

Differentially abundant taxa	22 DPI	38 DPI	**T test, *p* value**
f__Gemmatimonadaceae (uncultured)	more abundant in infected birds	-	$p < 0.05$
g__Butyricicoccus	more abundant in uninfected birds	-	$p < 0.05$
g__Collinsella	more abundant in uninfected birds	-	$p < 0.05$
g__Curtobacterium	more abundant in uninfected birds	-	$p < 0.05$
g__Domibacillus	more abundant in infected birds	-	$p < 0.05$
g__Faecalibacterium	more abundant in uninfected birds	-	$p < 0.05$
g__Paenibacillus	more abundant in uninfected birds	-	$p < 0.05$
g__Streptococcus	more abundant in infected birds	-	$p < 0.05$
g__Sutterella	more abundant in uninfected birds	-	$p < 0.05$
g__Vulcaniibacterium	more abundant in uninfected birds	-	$p < 0.05$
c__Blastocatellia (11-24)	-	more abundant in uninfected birds	$p < 0.05$
c__Holophagae (Subgroup_7)	-	more abundant in uninfected birds	$p < 0.05$
f__Carnobacteriaceae	-	more abundant in uninfected birds	$p < 0.05$
f__Gemmatimonadaceae (uncultured)	-	more abundant in uninfected birds	$p < 0.05$
f__Myxococcaceae	-	more abundant in uninfected birds	$p < 0.05$
f__Nitrosomonadaceae (Ellin6067)	-	more abundant in uninfected birds	$p < 0.05$
f__Nitrosomonadaceae (MND1)	-	more abundant in uninfected birds	$p < 0.05$
g__Anoxybacillus	-	more abundant in uninfected birds	$p < 0.05$
g__Blastococcus	-	more abundant in uninfected birds	$p < 0.05$
g__Domibacillus	-	more abundant in uninfected birds	$p < 0.05$
g__Gemmatimonas	-	more abundant in uninfected birds	$p < 0.05$
g__Nitrospira	-	more abundant in uninfected birds	$p < 0.05$
g__Sphingomonas	-	more abundant in uninfected birds	$p < 0.05$
g__Streptococcus	-	more abundant in uninfected birds	$p < 0.05$

g__Thermoactinomyces	-	more abundant in uninfected birds	$p < 0.05$
g__Vicinamibacteraceae	-	more abundant in uninfected birds	$p < 0.05$
o__Azospirillales (uncultured)	-	more abundant in uninfected birds	$p < 0.05$
o__Burkholderiales (TRA3-20)	-	more abundant in uninfected birds	$p < 0.05$
o__Solirubrobacterales (67-14)	-	more abundant in uninfected birds	$p < 0.05$
o__Vicinamibacterales (uncultured)	-	more abundant in uninfected birds	$p < 0.05$
p__Desulfobacterota (uncultured)	-	more abundant in uninfected birds	$p < 0.05$
p__Gemmatimonadota (AKAU4049)	-	more abundant in uninfected birds	$p < 0.05$
p__Myxococcota (bacteriap25)	-	more abundant in uninfected birds	$p < 0.05$
