# Supplementary figures and images for: Impact of Plasmodium relictum Infection on the Colonization Resistance of Bird Gut Microbiota: A Preliminary Study
Source: Pathogens. 2024 Jan 20;13(1):91. doi: 10.3390/pathogens13010091 (PMC10819382; doi:10.3390/pathogens13010091)

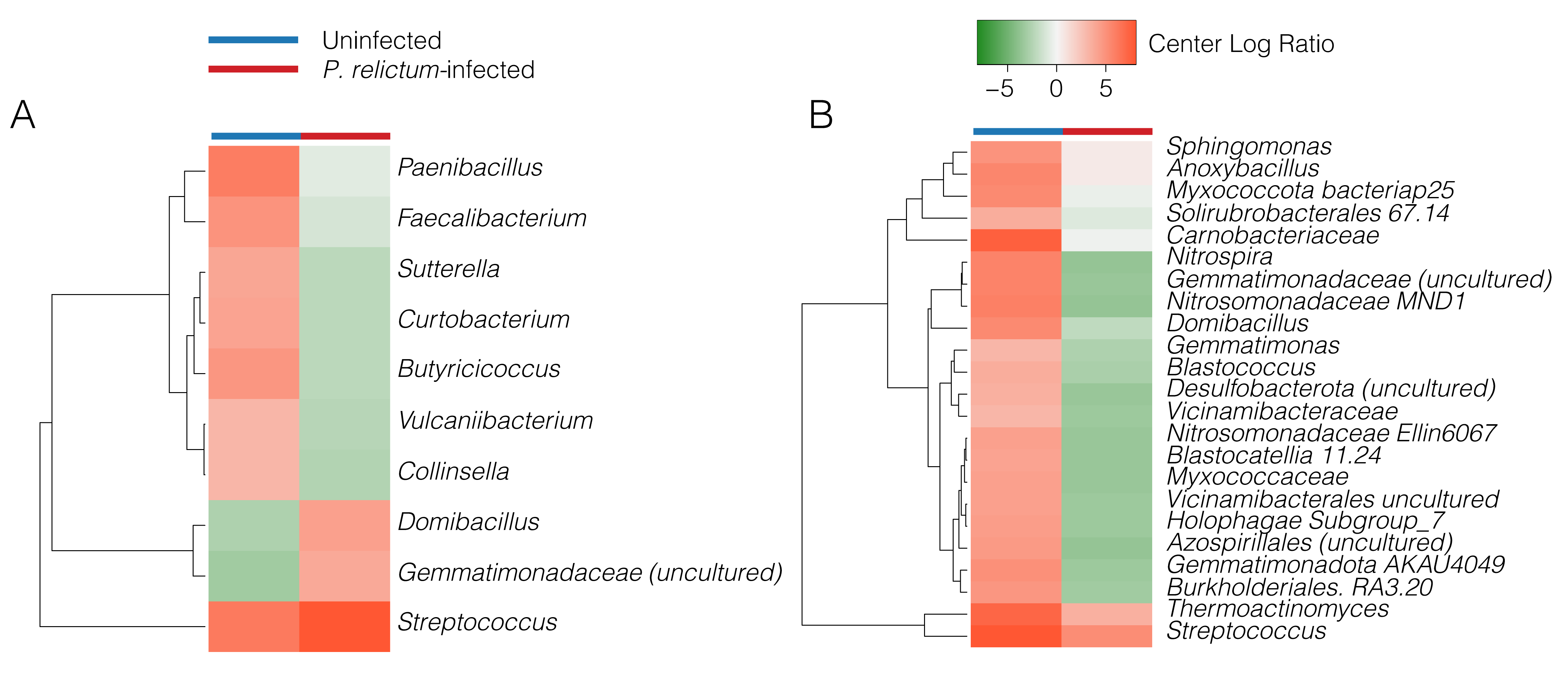

Supplement: Supplementary file 1 [file pathogens-13-00091-s001.zip › Supplementary figure S1.tif]

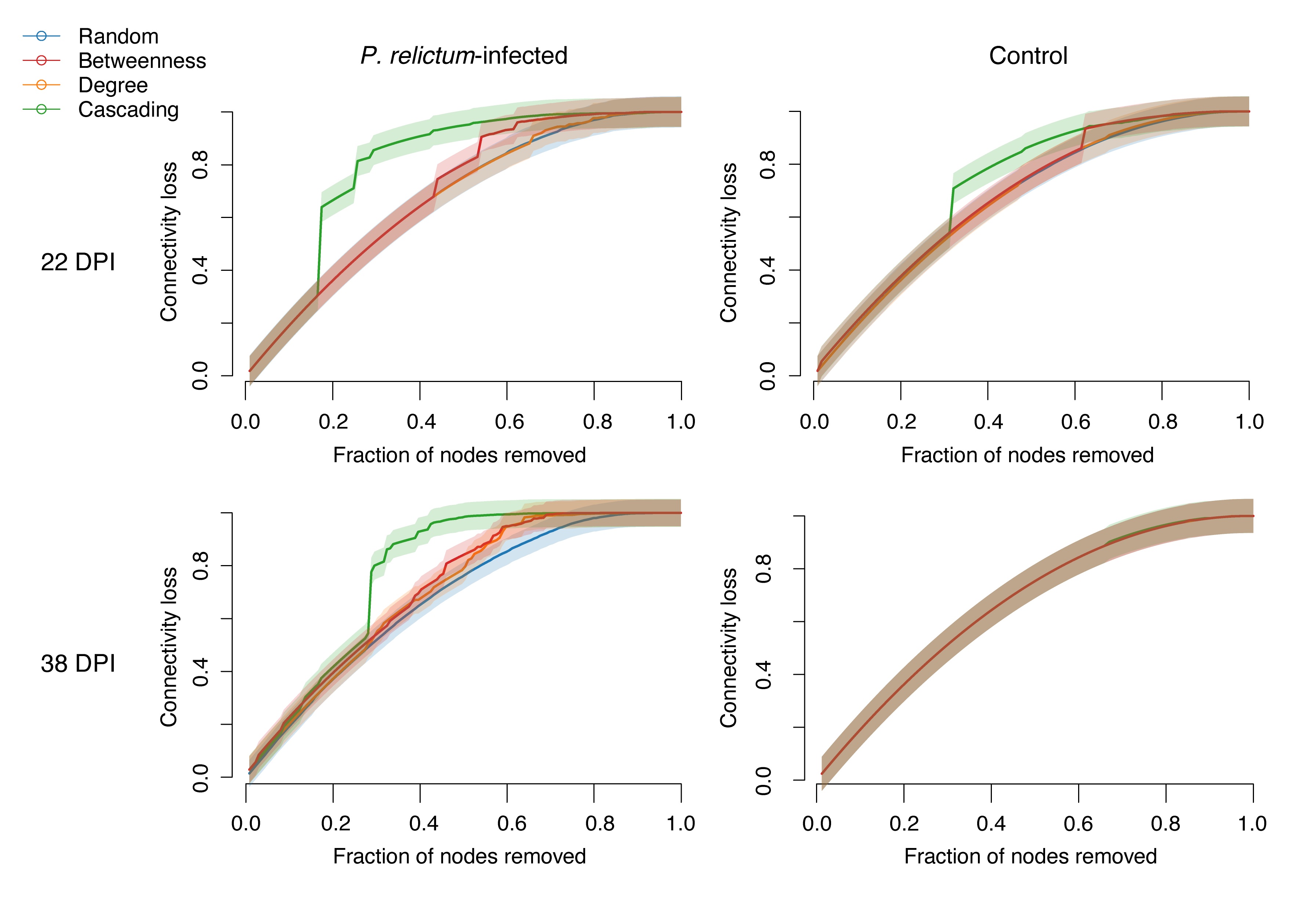

Supplement: Supplementary file 1 [file pathogens-13-00091-s001.zip › Supplementary figure S2.tif]
